# Advanced Practice Nursing: A Strategy for Achieving Universal Health Coverage and Universal Access to Health

**DOI:** 10.1590/1518-8345.1677.2826

**Published:** 2017-01-30

**Authors:** Denise Bryant-Lukosius, Ruta Valaitis, Ruth Martin-Misener, Faith Donald, Laura Morán Peña, Linda Brousseau

**Affiliations:** 1 PhD, Associate Professor, School of Nursing, McMaster University, Hamilton, ON, Canada.; 2 PhD, Professor, School of Nursing, Dalhousie University, Halifax, NS, Canada.; 3 PhD, Associate Professor, Daphne Cockwell School of Nursing, Ryerson University, Toronto, ON, Canada.; 4 PhD, Professor, Escuela Nacional de Enfermería y Obstetricia de la Universidad Nacional Autónoma de México, Ciudad de México, DF, Mexico.; 5 MSc, Nurse Practitioner (NP), Halton Region Health Unit, Oakville, ON, Canada.

**Keywords:** Advanced Practice Nursing, Delivery of Health Care, Primary Health Care

## Abstract

**Objective:**

to examine advanced practice nursing (APN) roles internationally to inform role development in Latin America and the Caribbean to support universal health coverage and universal access to health.

**Method:**

we examined literature related to APN roles, their global deployment, and APN effectiveness in relation to universal health coverage and access to health.

**Results:**

given evidence of their effectiveness in many countries, APN roles are ideally suited as part of a primary health care workforce strategy in Latin America to enhance universal health coverage and access to health. Brazil, Chile, Colombia, and Mexico are well positioned to build this workforce. Role implementation barriers include lack of role clarity, legislation/regulation, education, funding, and physician resistance. Strong nursing leadership to align APN roles with policy priorities, and to work in partnership with primary care providers and policy makers is needed for successful role implementation.

**Conclusions:**

given the diversity of contexts across nations, it is important to systematically assess country and population health needs to introduce the most appropriate complement and mix of APN roles and inform implementation. Successful APN role introduction in Latin America and the Caribbean could provide a roadmap for similar roles in other low/middle income countries.

## Introduction

Along with the development of stronger health systems in which primary health care is paramount, improved access to health and universal health coverage are recognized priorities for improving global health^1^ ^-^ ^2^. Following 60 plus years of global development, there is heightened recognition of advanced practice nurses and their impact on increased access to primary health care and improved quality of care and health outcomes^3^ ^-^ ^4^. At the intersection of primary health care and advanced practice nurses as two global phenomena is the concept of human resources for health. An adequate supply and appropriate mix of health care providers is critical to achieve the global agenda for health and advanced practice nurses are an essential component of country level health human resources^5^ ^-^ ^6^. Advanced practice nursing (APN) roles are at an early stage of development in Latin American countries^7^. Thus, there is tremendous opportunity to leverage these roles to achieve goals for access to health, universal health coverage and primary health care reform in these countries.

## Objectives

The purpose of this article is to examine what is known about the deployment and impact of APN roles internationally and to use this evidence to provide recommendations for developing these roles in Latin American countries to achieve universal health coverage and universal access to health. 

## Method

We examined literature related to APN roles, their global deployment, and APN effectiveness in relation to universal health coverage and access to health. The examination of APN roles was limited to the clinical nurse specialist (CNS) and the nurse practitioner (NP). 

The article begins with a summary of the WHO’s^2^ goals for universal health coverage and universal access to health in relation to primary health care and APN roles. Next the types and common features of APN roles are outlined, followed by an analysis of the global deployment of APN roles, including Latin America and the Caribbean. The effectiveness of APN roles is then examined in relation to universal health coverage and access to health. Implications for Latin American countries along with strategies to support effective APN role integration in primary health care are identified.

### Universal health coverage, universal access to health and advanced practice nursing roles in primary health care

Universal health coverage aims to strengthen health care delivery systems to promote access to care and improve health outcomes especially for disadvantaged populations. It involves the financing of health systems to ensure 1. the availability of comprehensive and high quality health services including promotion, prevention, treatment, rehabilitative and palliative care; and 2. equitable access to these services, regardless of social circumstances, without risk of financial hardship^2^. The right to health is an inherent value of universal health coverage. According to Margaret Chan, Director of the WHO^8^, universal health coverage, grounded in the delivery of integrated primary health care services, may provide the single most powerful tool for improving global health. The United Nations^9^ has reaffirmed its commitment to universal health coverage in new sustainability goals for 2030.

Although much progress has been made in Latin American countries to improve health care and implement varied financial models for universal health coverage, equitable access to health and essential primary health care remains elusive for millions of people in the region^10^ ^-^ ^14^. There are pressing needs to improve access to primary health care in rural communities and for services to improve health outcomes related to maternal and child mortality, infectious diseases, and aging. Augmented health promotion, prevention and management services are needed to reduce the burden and mortality associated with chronic conditions, especially for mental health, cancer, cardiovascular disease and diabetes.

Recognition of the need to strengthen primary health care worldwide dates back to the Declaration of Alma-Ata in 1978 and has been reinforced in subsequent policies such as the WHO Resolution WHA62.12^1^. However, it is only recently through two policy events that the need and opportunity to develop APN roles for primary health care in Latin America were formally identified. In August 2013, the WHO 2008-2012 progress report on nursing and midwifery^15^ ^)^ emphasized the need to develop specialized nursing and APN roles with the core competencies to meet population health and health services needs in revitalized primary health care systems. PAHO echoed this recommendation with Resolution CD 52.R13 to include advanced practices nurses as one part of an overall strategy to increase the primary health care workforce in Latin America^12^. Over the past two years nursing leaders from Latin America and the Caribbean have begun to explore strategies to support APN role development in their countries^7^ ^,^ ^16^ ^-^ ^19^. 

### Types of advanced practice nursing roles

The International Council of Nurses (ICN), defines an advanced practice nurse as a “registered nurse who has acquired the expert knowledge base, complex decision-making skills and clinical competencies for expanded practice, the characteristics of which are shaped by the context and/or country in which she/he is credentialed to practice. A master’s degree is recommended for entry level”^20^. Clinical practice involving the direct and indirect care of patients and their families, groups, communities or populations is the primary focus of APN roles. In addition to clinical practice, other APN role responsibilities include the education of nurses and other health professionals, evidence-based practice and research, organizational leadership and professional development^21^ ^-^ ^23^. It is the combined effect of these multi APN role responsibilities that leads to innovation and health care improvement.

The impetus for APN roles is contextually driven at the country and/or organizational level. Internationally, this has resulted in some confusion about what constitutes the role and a plethora of role titles. One international survey identified 52 different APN role titles such as the clinical nurse specialist (CNS), nurse practitioner (NP), advanced practice nurse, nurse specialist, nurse consultant, nurse midwife, and nurse anesthetist^24^. Of these role titles, CNS and NP are the most common^25^ ^-^ ^26^. Regardless of the type, common characteristics of APN roles have been identified including: completion of an accredited education program designed to produce advanced practice nurses and formal licensure, registration, certification and credentialing^21^. Depending on the country-specific regulatory mechanisms, advanced practice nurses may have an expanded scope of practice with title protection and the legal authority to diagnose, prescribe medications and treatments, refer patients to other health professionals, and admit patients to hospital. How CNSs and NPs implement their roles is highly variable and dependent on population health and health setting needs. In general, CNSs have an in-depth knowledge of a specialized area of nursing practice and have the same scope of practice as a registered nurse. In addition to patient care they have heightened responsibilities for nursing and health systems improvement such as providing leadership and education and promoting evidence-based practice^27^. Nurse practitioners have an expanded scope of practice that usually includes advanced health assessment, illness and injury prevention and therapeutic management and tend to spend more of their time providing direct patient care^22^.

### Global deployment and use of advanced practice nursing roles

The actual extent of global APN role deployment is not known. The introduction of APN roles is at different stages of development across countries and inconsistent mechanisms for regulating and thus identifying nurses in the role, make it difficult to monitor and track practice patterns. According to the ICN^28^, 70 countries have or are interested in introducing APN roles. An international survey documented APN roles in at least 38 countries^24^. The most established APN roles are found in high income countries such as the United States, Canada, United Kingdom and Australia^25^. In the last decade there has been further expansion of APN roles, especially in high income countries in Europe, Africa, Asia and the Middle East^29^ ^-^ ^32^. There are few reports of APN role development in low and middle income countries.

Improving health outcomes and increasing access to health care in rural and remote communities and for vulnerable populations (e.g., homeless, mental health, drug addiction) in urban communities have been initial drivers for the introduction of NPs in primary health care settings^33^ ^-^ ^34^. Initially, CNSs were introduced to keep pace with advances in treatment and technology and the increased complexity of nursing care for specialized populations in acute care settings^35^. More recently, CNSs and NPs are also being deployed internationally to a broad range of community, long-term care and acute care settings as catalysts for improving health outcomes and quality of care and innovation to deliver more sustainable models of health care^12^ ^,^ ^15^ ^,^ ^25^ ^,^ ^36^ ^-^ ^40^. Current issues driving new care delivery models and the introduction of APN roles include aging populations and care of the elderly, needs for enhanced health promotion and chronic disease prevention and management, primary health care reform, health workforce shortages and escalating health care costs. 

APN roles are at an early stage of development in Latin America and the Caribbean^16^ ^-^ ^17^ ^,^ ^41^. There are few established APN education programs and regulatory mechanisms for APN roles do not yet exist, but are under development in some countries. Of these countries, Jamaica may have the most experience in relation to advanced practice with the introduction of the family, pediatric and mental health/psychiatric NP education programs in 1977 and 1978^42^ ^-^ ^46^. Factors facilitating the introduction of the NP role were the government’s policy agenda for primary health care reform and recognition of the NP’s complementary curative and health promotion skill sets, and shortages of physicians in rural areas and underserviced communities. Currently in Jamaica, the University of West Indies offers CNS, family NP and mental/health/psychiatric NP programs at the Master’s degree level. Published reports on APN roles in other Latin American or Caribbean countries are limited, especially related to primary health care. In Brazil, a CNS role in pediatric oncology has been established^47^. In Chile, the University of Los Andes has an advanced practice nursing program for CNSs in adult critical care (http://postgrados.uandes.cl/mpae/). 

At this time, Latin American countries at the greatest state of readiness to introduce APN roles for primary health care are Brazil, Chile, Colombia and Mexico. Nursing leaders from these countries recently met with their counterparts from Canada and the United States to determine strategies for development of APN competencies and curricula^17^. A master’s degree is recommended as the foundation for APN education^20^. All four countries have existing graduate nursing education programs from which APN education programs can be built. Brazil in particular, is well positioned to establish APN education programs with 51 master’s and 36 doctoral nursing education programs^48^. Brazil also distinguishes two streams of master’s degree education; academic and professional. Academic programs are designed to produce nursing researchers and faculty, while professional programs are designed to develop nurses working in varied higher level roles in the health care system. At least 15 Professional Master’s Degree programs exist in Brazil. Their focus on developing applied knowledge and skills may make these programs amenable for adaptation to APN education programs^49^. In Chile and Mexico, partnerships with NP education programs at universities in the United States have stimulated the development of APN education programs. In Colombia, stakeholder engagement activities between the Ministry of Health and academic nursing and policy leaders is setting the stage for developing APN education programs^17^. 

Barriers to the introduction of APN roles in Latin America and Caribbean countries are similar to those reported in the international literature including role clarity, legislation, regulation, education programs and resources, funding, and physician resistance^16^ ^,^ ^19^ ^,^ ^24^ ^-^ ^25^ ^,^ ^50^ ^-^ ^51^. In relation to role clarity, nursing leaders from Latin America and the Caribbean identify a general lack of awareness and understanding of APN roles within the nursing profession and amongst health care policy decision-makers within governments^16^. At the political and policy level, a shared challenge across all countries has been legislative barriers to define, legitimize and facilitate regulation of nurses with expanded scopes of practice. There is also need to strengthen nursing education in Latin America to improve access to standardized, high quality programs including those specific to APN^16^ ^-^ ^17^ ^,^ ^42^. There are shortages of nursing faculty and few faculty with the expertise to develop and teach in clinically-focused APN education programs. A unique aspect of the Latin American and Caribbean region is the mix of low, lower middle, upper middle and high income countries resulting in different health care needs and economic capacity to support APN education and subsequent roles in practice^52^. Diverse strategies will be required to introduce APN roles and curricula that address the heterogenic cultural, geographic, socioeconomic and political contexts of member countries. 

Other challenges for introducing APN roles for primary health care in most Latin American countries is a general shortage of nurses and a high proportion of technically trained nurses (e.g., nursing aides) compared to degree prepared professional nurses in the workforce to draw on for further development^11^ ^,^ ^13^ ^,^ ^16^ ^,^ ^42^ ^,^ ^53^. There is also limited focus on community or primary health care in nursing education programs and lack of recognition among nurses of primary health care as an area of specialization or a desired career path. The lack of existing labor market positions for advanced practices nurses may also be a deterrent to enrolment in APN education programs and result in underemployment and frustration for program graduates. While most countries have an overall shortage of health care providers, they have a greater number of physicians compared to nurses in the workforce. Physician resistance and the over medicalization of health care, in which nurses are undervalued, are perceived barriers to introducing APN roles in Latin America and other countries^11^ ^,^ ^16^ ^,^ ^24^. 

### Alignment of advanced practice nursing roles with the global agenda for improving universal health coverage and access to health

Universal health coverage is dependent on adequate financing which is difficult to achieve for all countries, but especially those with low and middle incomes, due to rising health care costs. In Latin America and the Caribbean, inadequate financing of universal health coverage in some countries has contributed to a mix of public and private insurance plans, high out-of-pocket expenses and inequitable access to timely and high quality care for the unemployed, poor and vulnerable populations^10^. Improved financing to provide comprehensive universal health coverage is an important issue for the region, where over 25% or 130 million people live in chronic poverty^54^. 

One strategy is to offset the costs of financing universal health care coverage by gaining efficiency in health care. The WHO has provided ten recommendations for reducing the 40% of health care spending that is wasted through inefficiency^55^. At least five of these ten recommendations could be addressed through the introduction of APN roles, not just in primary health care, but across the health system where needs and inefficiencies exist. These five recommendations are related to 1. the overuse of health care services, 2. inappropriate and costly staff mix and unmotivated workers, 3. inappropriate hospitalization and length of stay, 4. errors and suboptimal quality of care and 5. inefficient mix or level of health care interventions. 

Multiple systematic reviews have demonstrated that CNSs and NPs are safe and effective health care providers. In relation to health care service use, CNSs and NPs may achieve cost savings through: shorter hospital lengths of stay and reduced hospital readmissions for the elderly and patients transitioning from hospital to home^40^ ^,^ ^56^ ^-^ ^58^; fewer tests and reduced clinic, physician and emergency department visits for cancer patients^59^; and lower consultation costs for patients in primary care^3^ ^-^ ^4^. 

Relevant to appropriate staff mix, as substitutes for other providers (usually physicians) to address workforce shortages, CNSs and NPs achieve equal or better health outcomes and satisfaction with care in inpatient and outpatient settings and for transitional care^3^ ^,^ ^38^ ^,^ ^40^ ^,^ ^56^ ^,^ ^59^ ^-^ ^60^. These same systematic reviews also demonstrate the benefits of the complementary addition of CNS and NP roles to health care teams to improve patient health outcomes, satisfaction with care and quality of care. In studies of team-based models of primary health care, the addition of an NP increases access to health promotion and prevention services to meet community needs^61^ ^-^ ^63^ and improves the quality of care for chronic disease management^64^. 

Other studies indicate that CNSs help support the development of a motivated workforce by promoting staff satisfaction^65^ and facilitating the recruitment and retention of high quality nurses^66^. They also reduce errors and suboptimal care by promoting patient safety and preventing complications^58^ ^,^ ^67^ ^-^ ^68^ and lower the use of inefficient or ineffective health care interventions by promoting provider and patient uptake of best practices^69^ ^-^ ^70^. 

Compared to usual care, CNSs and NPs also improve access to health by achieving better health outcomes for a broad range of patient populations in varied practice settings. Older adults receiving APN care in ambulatory care for dementia or chronic heart failure or those in long term care settings have reduced mortality rates and improved health outcomes related to depression, aggressive behavior, incontinence, and pressure ulcers^36^ ^,^ ^57^. In primary care and in ambulatory care settings, NP care for patients with chronic conditions such as heart disease, hypertension, and diabetes results in better indicators of disease control such as lower blood pressure, reduced serum cholesterol levels, and reduced glycated hemoglobin^3^ ^-^ ^4^. CNS outpatient care is associated with improved mental health for patients with psychiatric problems, better disease control and quality of life for patients with heart failure, and reduced symptoms of disease activity for patients with arthritis^38^. CNS transitional care also permits earlier hospital discharge of high risk patient populations (cancer, pregnancy, older adults, heart failure, neonates) while at the same time achieving equivalent or better health outcomes^40^. Examples of improved outcomes include increased survival for patients with advanced cancer or heart failure and improved immunization rates for very low birth weight infants^40^. Similar findings of equal or better health outcomes for NP transitional care compared to usual care have been demonstrated for patients undergoing gynecological surgery and those with complex conditions, asthma, or myocardial infarction^56^. Across these studies, aspects of CNS and NP care are thought to contribute to better health outcomes include the delivery of patient-centred care, tailored patient education and coaching, improved continuity and coordination of care and collaboration with other health professionals. Improved patient self-care and uptake of healthy life style behaviors are also a consequence of CNS and NP care that may lead to improved health. 

### Implications for the implementation of advanced practice nursing roles in Latin American and Caribbean countries

If the process is done well, the introduction of APN roles in Latin America and the Caribbean could provide a roadmap or template for introducing these roles in low and middle income countries where few such roles exist. To achieve optimal impact for improving universal health coverage and access to health, a systematic approach to APN role introduction is required to determine patient populations and communities with unmet health and health service needs and where the greatest gains in health outcomes, health care efficiency and health systems improvement can be made^55^. Such an approach is offered by the PEPPA Framework which outlines a Participatory, Evidenced-based and Patient-Centred Process for designing, implementing and evaluating APN roles^71^. PEPPA has been implemented in at least 16 countries for a broad range of patient populations in various settings and is recognized as best practice for introducing APN and other advanced health provider roles^72^ ^-^ ^73^. A framework strength is the early and ongoing involvement of representatives from influential stakeholder groups including patients, physicians and other health providers, regulators, educators, health care administrators and government policy makers. Through stakeholder engagement and needs assessment strategies, agreement on priority population health and health system needs and goals to be address by the APN role can be established. 

The framework also applies principles for effective health human resource planning to determine the type of APN role, the optimal complement and mix of other health care providers, and how the role will interact with other health care team members to achieve identified goals and related outcomes^73^. Due to the diversity of health systems, funding arrangements, human resources, and population health needs it will be important for each country in the Latin American and Caribbean region to conduct its own systematic process for prioritizing and defining the APN role or roles to be introduced^11^. 

Given the overall shortage of nursing and other provider roles in primary health care in most countries, it will be important to consider the introduction of APN roles within the context of creating a primary health care workforce^74^ ^-^ ^75^. The introduction of APN roles can be leveraged to improve nursing workforce outcomes by promoting primary health care as a recognized and desirable career for nurses. For example, establishing a career ladder with level competencies from novice to advanced nursing expertise can be used to create clearly defined role positions and salary grades; provide the basis for certification and credentialing; inform undergraduate, graduate, and continuing education programs; guide career reviews and performance evaluations; and create a sufficient pool of nurses for faculty development^76^. Further, the selection and design of APN roles should include relevant competencies and expectations to support the development of primary health care nurses at the point of care. Examples include APN role responsibilities for educating nurses to develop their clinical, evidence-based practice and leadership skills; coaching and mentoring to build their confidence; acting as clinical faculty and preceptors for undergraduate and graduate students; providing consultation and support to manage complex patient care situations; and facilitating the use of technology for education and care delivery^5^. APN leadership is also required to advocate for policies to support nursing practice in new care delivery models providing a better balance between health promotion and prevention and disease-focused care^11^ ^,^ ^75^. APN leadership can also be used to support healthy workplace environments for nurses and other health care providers. Leadership strategies may include managing and developing interprofessional teams, promoting effective interprofessional team work, and supporting the developing of health professionals and community health workers^77^ ^-^ ^78^. 

The PEPPA framework also integrates planning steps to identify potential barriers and optimize enablers to effective APN role implementation related to stakeholder and APN education, recruitment and retention, and health care policies including legislation and regulation^71^. Important enablers are public awareness of the role and strong nursing leadership across multi-sectors of the health care system to advocate for systems changes to support APN role implementation. Nursing and intersectoral leadership and partnerships will be required to obtain dedicated funding for the role and other support structures that align with health care priorities^41^. Addressing physician concerns about APN role impact on their income and liability issues and role clarity to support effective intra- and inter-professional collaboration among nurses, physicians and other providers is also essential for effective role implementation^5^. 

As outlined above, there are a multitude of systematic reviews of the international literature, predominately from high income countries, confirming that CNSs and NPs are safe and effective health care providers. Within the context of APN role introduction in Latin America and the Caribbean, research and the use of other evaluation methods will be required to ensure the effective use, optimal implementation and long-term sustainability of these roles. A major challenge in some Latin American countries is the lack of good baseline workforce and health care system data to inform the introduction and design of APN roles and to support subsequent evaluations^41^. To address country decision-making needs for better and more contextually relevant data, the PEPPA framework has been enhanced to provide detailed guidance for APN role evaluation^79^. The enhanced framework provides examples of evaluation questions and methods to generate relevant country data to support health care redesign involving the introduction of APN roles, assess the effectiveness of role implementation strategies, and to determine the impact of the roles. Use of the framework will help countries in Latin America and the Caribbean to map out a detailed APN role evaluation plan with identified priorities, timelines, methods, and resources. 

## Conclusions

There is a substantive body of international evidence about the positive impact of APN roles for improving patient health outcomes, quality of care and health system efficiency. The implementation of these roles can address country needs to improve universal health coverage and universal access to health in Latin America and the Caribbean. Several middle and high income Latin American countries with access to graduate nursing education are posed to introduce these roles. Other important elements in place to support APN role introduction in these countries include the alignment of APN outcomes with health care policies for primary health care reform and a developing coalition of nursing leaders across health care, academic, and health policy sectors both within and external to Latin American countries. Further expansion to engage other intersectoral leaders will be required to move the APN agenda forward.
